# Differential Induction of Type I and Type III Interferons by Swine and Human Origin H1N1 Influenza A Viruses in Porcine Airway Epithelial Cells

**DOI:** 10.1371/journal.pone.0138704

**Published:** 2015-09-18

**Authors:** Venkatramana D. Krishna, Erin Roach, Nathan A. Zaidman, Angela Panoskaltsis-Mortari, Jessica H. Rotschafer, Scott M. O’Grady, Maxim C-J. Cheeran

**Affiliations:** 1 Department of Veterinary Population Medicine, College of Veterinary Medicine, University of Minnesota, St. Paul, Minnesota, United States of America; 2 Department of Animal Science, University of Minnesota, St. Paul, Minnesota, United States of America; 3 Department of Pediatrics, University of Minnesota, Minneapolis, Minnesota, United States of America; University of Saskatchewan, CANADA

## Abstract

Interferons (IFNs) have been shown to inhibit influenza A virus (IAV) replication and play an essential role in controlling viral infection. Here we studied the kinetics and magnitude of induction of type I and type III IFN transcripts by primary porcine airway epithelial cells (pAECs) in response to swine and human origin IAV. We observed that swine influenza viruses (SIV) replicate more efficiently than the human pandemic influenza A/California/2009 (pH1N1 CA/09) in pAECs. Interestingly, we also found significant difference in kinetics of IFN-β, IFN-λ1 and IFN-λ3 gene expression by these viruses. While there was delay of up to 12 hours post infection (h p.i.) in induction of IFN genes in pAECs infected with swine IAV A/Sw/Illinois/2008 (H1N1 IL/08), human pH1N1 CA/09 rapidly induced IFN-β, IFN-λ1 and IFN-λ3 gene expression as early as 4 h p.i. However, the magnitude of IFN-β and IFN-λ3 induction at 24 h p.i. was not significantly different between the viral strains tested. Additionally, we found that swine H1N1 IL/08 was less sensitive to dsRNA induced antiviral response compared to human pH1N1 CA/09. Our data suggest that the human and swine IAVs differ in their ability to induce and respond to type I and type III interferons in swine cells. Swine origin IAV may have adapted to the pig host by subverting innate antiviral responses to viral infection.

## Introduction

Influenza A virus (IAV) is a common respiratory pathogen infecting many different hosts including pigs, humans, and birds. Although influenza viruses have co-evolved with their respective hosts, they are capable of transmitting infection between species [1]. Swine respiratory epithelial cells express both α2,6,- and α2,3,- linked sialic acids, the receptor determinants for human and avian influenza viruses respectively [2]. Consequently, pigs are susceptible to infection with IAV of human and avian origin, in addition to swine influenza viruses (SIV), increasing the possibility that pigs serve as ‘mixing vessels’ for the generation of reassortant viruses with pandemic potential [3]. Although IAV of human and avian origin can cross the species barrier and infect pigs, fitness of these viruses are not equal among species. For e.g. titers obtained from infection with human and avian origin viruses in pigs were reported to be lower than with SIV [4]. It has been demonstrated that the triple reassortant H3N2 IAV has higher infectivity in pigs compared to human lineage H3N2 virus. The phenotypes of these viruses related to replication and infectivity in swine respiratory epithelial cells were shown to be dependent on properties of the HA gene [5]. The differences in the levels of infectivity of H3N2 viruses were attributed to the differences in binding affinities of the virus to sialic acid residues in swine respiratory epithelial cells [6].

Virus infectivity depends not only on viral genetic factors but also on its ability to evade host antiviral responses. Type I and type III interferons, the components of innate immune responses, are rapidly induced during viral infection and play a critical role in the antiviral response [7, 8]. Type III IFNs, first discovered in 2003, include three proteins; IFN-λ1 (IL-29), IFN-λ2 (IL-28A) and IFN-λ3 (IL-28B) [9, 10]. Both type I and type III IFNs activate the same signaling pathway, leading to the induction of IFN-stimulated genes (ISGs) [11–13]. Toll like receptors (TLR3 and TLR7) and retinoic acid inducible gene-1 (RIG-1) are involved in activating IFN production, although RIG-1 pathway is the major cytosolic IAV recognition pathway in epithelial cells [14, 15]. Activation of RIG-1 by double stranded RNA (dsRNA) [16] activates intracellular signaling that leads to expression of IFNs in infected cells. The IFNs produced by virus infected cells activate an antiviral state in surrounding uninfected cells. Notably, many viruses including IAV evolved to inhibit production and function of these IFNs as a fitness mechanism to evade innate host responses [7].

IAV achieves evasion of the host IFN system via the selective binding properties of the NS1 protein, which inhibits type I IFN synthesis by multiple mechanisms. Firstly, IAV NS1 binds to and sequesters dsRNA formed during replication [17, 18], thus preventing activation of dsRNA induced oligoadinylate synthetase (OAS) and protein kinase R (PKR)[19]. In addition, NS1 binds to single stranded viral RNA bearing uncapped 5’ phosphates [20] which ‘masks’ the virus from recognition by RIG-1. Finally, NS1 interacts with RIG-1 to inhibit downstream signaling [21, 22] by directly binding to and blocking PKR activation [23]. These evasion mechanisms by viral NS1 proteins likely co-evolved with viruses in their respective hosts, thus providing a significant replicative advantage for the maintenance and survival of IAV within the host population (for review see/ Hale BG, et al 2008) [24].

Epithelial cells of the respiratory tract are the primary targets of influenza viruses. Porcine airway epithelial cells (pAEC) provide a valuable *in vitro* model to study the cellular tropism and host innate responses to influenza virus infection [5]. To evaluate viral tropism and porcine anti-viral responses to human and swine origin IAV strains, primary pAECs were isolated from healthy neonatal pigs and immortalized by transfection with human Telomerase Reverse Transcriptase (hTERT) using lentiviral vector. Immortalized cells maintain their epithelial cell phenotype and had comparable properties to primary pAECs. The major objective for the present study was to determine if differences in porcine interferon responses to IAV strains contribute to viral fitness in pig cells. We observed that titers of swine IAV isolates were significantly higher in primary pAECs compared to the human pandemic IAV. We hypothesize that differences in kinetics and magnitude of IFN induction by porcine cells in response to swine and human IAV contribute to differential susceptibility to these viruses. This hypothesis was tested by studying type I and type III IFN expression at transcript level in primary pAECs following infection with swine H1N1 IL/08 and human pH1N1 CA/09 and evaluating the ability of these viruses to counteract poly I:C induced antiviral responses by pAECs.

## Materials and Methods

### Ethics Statement

All experiments in this study using animals were conducted under protocols approved by the University of Minnesota Institutional Animal Care and Use Committee and in accordance with the Guide for the Care and Use of Laboratory Animals.

### Cells and viruses

Madin-Darby canine kidney (MDCK) cells free of *Mycoplasma* were obtained from ATCC (ATCC CCL-34) and maintained in Dulbecco’s modified Eagle medium (DMEM) supplemented with 2% heat inactivated fetal bovine serum (FBS), 100 U/ml penicillin G and 100 μg/ml streptomycin and 1mM sodium pyruvate. Absence of *Mycoplasma* contamination was confirmed using MycoAlert mycoplasma detection kit (Lonza, Walkersville, MD) according to manufacturer’s recommendations. The swine influenza viruses; A/Sw/Illinois/2008 (H1N1 IL/08), A/Sw/Iowa/2004 (H1N1 IA/04), and A (H3N2) variant virus (H3N2v), and the human pandemic influenza A/California/04/2009 (pH1N1 CA/09) were obtained from the University of Minnesota Veterinary Diagnostic Laboratory (St Paul, MN). Viruses were propagated in MDCK cells in DMEM containing 0.5 μg/ml TPCK-trypsin (Worthington Biochemical Corporation, Lakewood, NJ) and purified from the clarified cell culture supernatants by ultracentrifugation through a 30% (w/v) sucrose cushion and stored in aliquots at -80^o^ C. Culture supernatant from un-infected MDCK cells were processed similarly to use for mock infection.

### Isolation of primary pAECs

Primary pAECs were isolated from three healthy neonatal pigs as described previously [5] with slight modifications. Three day old pigs were transported to the laboratory from the farm and euthanized using Fatal-Plus^®^ solution (Vortech Pharmaceutical Ltd, Dearborn, MI) given intravenously. Trachea and associated airway tissue were harvested under aseptic conditions inside a Class II A2 biosafety cabinet. Trachea were washed in phosphate buffered saline (PBS), placed in 25 ml of tissue dissociation medium (Mg^++^ and Ca^++^ free Hanks balanced salt solution (HBSS) supplemented with 100 U/ml penicillin G, 100 μg/ml streptomycin, 100 μg/ml gentamicin, 25 mM HEPES and 1.4 mg/ml pronase) and incubated at 4°C for 48 h. Fetal bovine serum (FBS) was then added to a final concentration of 10% to terminate protease activity and the cells were collected by centrifugation at 250 x g for 8 minutes at 15°C. The pellet was resuspended in pAEC growth medium (DMEM/F12 medium supplemented with 10% FBS, 100 U/ml penicillin G, 100 μg/ml streptomycin, 100 μg/ml gentamicin, 25 mM HEPES, 1X nonessential amino acids, 5μg/ml amphotericin B, 1% epidermal growth factor (EGF) and 5.5 μg/ml recombinant human insulin), seeded into uncoated tissue culture flask and incubated at 37°C /6% CO_2_ for 12 to 24 h to remove fibroblasts. The non-adherent epithelial cells were pelleted by centrifugation, resuspended in pAEC growth medium and seeded into type VI collagen coated flasks. The cells were grown at 37°C /6% CO_2_ until 100% confluent with medium change every 2 days.

### Immortalization of pAECs

Primary cells were transfected with hTERT contained within a lentiviral vector (Applied Biological Materials, Richmond, BC). The cells were maintained in 6-well culture plates and transfection was initiated at 70% confluency. Culture media was replaced with a 1:1 dilution of media and Lenti-hTERT stock solution in the presence of 5μg/mL polyprene and incubated overnight. The following day the virus containing media was aspirated and replaced with fresh culture media for 72 h. Cells were washed with PBS and passaged using Trypsin-EDTA (0.05%) (Life Technologies, Carlsbad, CA). Selection was performed by resuspending cells in fresh culture media in the presence of 4 μg/mL puromycin. Puromycin resistant pAECs were expanded into a homogenous cell line after 15 days in culture.

### Immunofluorescence staining

pAECs were grown on type VI collagen coated microscope slides in 100mm tissue culture dish. When the monolayer is about 70% confluent, cells were fixed with cold methanol for 10 minutes at -20°C. After washing with 0.5% Triton-X 100 in PBS, the cells were treated with blocking solution consisting of 1% BSA, 10% normal goat serum and 0.5% Triton-X 100 in PBS at room temperature for 30 minutes. The slides were subsequently incubated at 4°C overnight with 1:100 diluted monoclonal anti-cytokeratin 18 antibody (Pierce Thermo scientific, Rockford, IL) followed by 1:500 diluted goat anti mouse IgG1-Alexa Fluor 488 (Life technologies, Eugene, OR) at room temperature for 30 min. The cells were counterstained with 4’,6-diamino-2-phenylindole dihydrochloride (DAPI) and examined under Nikon ECLIPSE Ci fluorescent microscope (Nikon instruments Inc, Melville, NY).

### Measurement of transepithelial resistance

Transepithelial electrical resistance (TER) was measured on confluent, primary, and immortalized pAECs grown on Snapwell polyester membranes under liquid-liquid interface conditions. Measurements were made using a Ag-AgCl “chopstick” electrode coupled to a EVOM epithelial voltohmmeter (World Precision Instruments, Sarasota, FL) and were recorded over a time period of 50 days.

### IAV infection and growth kinetics

pAECs were grown in 6-well tissue culture plates (Corning Inc, Durham, NC). When the monolayer was 80% confluent, cells were washed twice with Hank’s balanced salt solution (HBSS) and infected with IAV at multiplicity of infection (MOI) of 0.01 in pAEC growth medium without FBS. Trypsin was not added during infection since our preliminary studies indicated similar levels of virus replication in pAECs in the presence or absence of exogenous trypsin (data not shown). After 1 h of virus adsorption, the inoculum was removed, cells were washed twice with HBSS and allowed to grow in pAEC growth medium containing 2% FBS. At 0, 4, 8, 12, 24, 48, 72, and 96 h p.i., 0.1 ml of culture medium was collected for analysis of virus titer by TCID_50_ assay. TCID_50_ assay was performed in MDCK cells in media containing 0.5 μg/ml TPCK-trypsin (Sigma, St. Louis, MO). The limit of detection of TCID_50_ assay is 6.56 x 10^1^ TCID_50_/ml (Log_10_ TCID_50_/ml = 1.82).

For analysis of IFN gene expression, pAECs were infected with IAV at 1 MOI or mock infected. At 4, 8, 12, and 24 h p.i. cells were harvested in RLT lysis buffer (Qiagen, Valencia, CA) for total RNA extraction.

### Virus binding to the cells and flowcytometry

Virus binding assay was performed using flowcytometry as described previously [25] with minor modifications. pAECs (10^5^ cells) were pre-chilled on ice for 30 minutes prior to infection with IAV at an MOI of 50. Virus was incubated with pAECs for 90 minutes on ice, to allow virus binding but prevent endocytosis of viral particles. After incubation, cells were washed three times with cold DMEM to remove any unbound virus. To control for possible internalized virus, cells were washed with acid glycine (0.1M Nacl, 0.1M glycine; pH: 3.0) for 1 min to remove cell surface bound virus from the cells. pAECs were fixed with 4% paraformaldehyde in PBS, permeabilized with 0.1% Triton X-100 and treated with blocking buffer containing 2% FBS, 5% normal goat serum in PBS for 30 min at 4°C. To detect bound virus, cells were incubated with monoclonal antibody to influenza A nucleoprotein (NP) (MAB8800; EMD Millipore corporation, Temecula, CA) for 30 min. followed by Alexa Fluor 647 conjugated goat anti-mouse IgG2a (Molecular probes Inc, Eugene, OR) for 30 min at 4^o^ C. The MAB detects NP from all IAV strains. Mouse IgG2a isotype (eBioscience, San Diego, CA) was used to control for non-specific/background fluorescence. Labeled cells were analyzed on a BD FACSCanto flowcytometer equipped with BD FACSDiva software (BD Biosciences, San Jose, CA). Data were analyzed using FlowJo software (Tree Star Inc, Ashland, OR).

### Quantitative RT-PCR

Total RNA was extracted from virus-infected or mock-infected cells using RNeasy plus mini kit (Qiagen, Valencia, CA) per manufacturer’s instruction. Genomic DNA was removed by gDNA eliminator spin column supplied in the kit. The purity of RNA was assessed by calculating the ratio of OD260/OD280. 1 μg of total RNA was reverse transcribed using Tetro cDNA synthesis kit (Bioline, Taunton, MA) according to manufacturer’s recommendations. The cDNA was amplified by RT-qPCR using SensiMix SYBR No-Rox kit (Bioline, Taunton, MA) in LightCycler 480 II (Roche diagnostics GmbH, Germany). The cycling conditions were as follows: 95^o^ C for 10 min followed by 45 cycles of 95^o^ C for 15 sec, 55^o^ C for 30 sec and 72^o^ C for 15 sec. The specificity of qPCR was assessed by analyzing the melting curves of PCR products. Ct values were normalized to β-actin gene or porcine GAPDH (for hTERT) to obtain ∆Ct and the difference between the ∆Ct values of the virus-infected sample and that of the mock-infected sample was calculated as ∆∆Ct. The fold change was expressed as 2^-∆∆Ct^ as previously described [26]. The sequences of primers used and the expected amplicon size are shown in Table 1.

**Table 1:** Sequences of primers used in RT-qPCR.

Primer name		Sequence (5’→ 3’)	Amplicon size (bp)
IFN-α1	forward	CAGTTCTGCACTGGACTGGA	169
	reverse	CACAGGGGCTGTAGCTCTTC	
IFN-β	forward	TGCAACCACCACAATTCC	79
	reverse	CTGAGAATGCCGAAGATCTG	
IFN-λ1	forward	TGTCACCACAGGAGCTGAAG	161
	reverse	TAGCTCAGCCTCTAAGGCCA	
IFN-λ3	forward	TTGGCCCAGTTCAAGTCTCT	101
	reverse	GAGCTGCAGTTCCAGTCCTC	
Mx1	forward	TAGGCAATCAGCCATACG	80
	reverse	GTTGATGGTCTCCTGCTTAC	
OAS1	forward	TCCACTCCCCTCCCGACTC	133
	reverse	GGCTTCCTTGACCTGTGTTCG	
hTERT	forward	GAGAACAAGCTGTTTGCGGG	225
	reverse	GGCCGGCATCTGAACAAAAG	
β-actin	forward	CACGCCATCCTGCGTCTGGA	380
	reverse	AGCACCGTGTTGGCGTAGAG	
pGAPDH	forward	CAAGAAGGTGGTGAAGCAGG	167
	reverse	ACCAGGAAATGAGCTTGACG	

### Treatment of cells with poly I:C or conditioned medium from poly I:C treated pAECs

pAECs were treated with 50 μg/ml polyinosinic- polycytidylic acid (poly I:C) (Sigma, St. Louis, MO) or saline (vehicle). After 24 h, the cells were infected with IAV at 0.01 MOI. At 24 h p.i. culture supernatants were collected for analysis of virus titer by TCID_50_ assay. To prepare poly I:C pAEC conditioned medium, pAECs were treated with 50 μg/ml poly I:C for 24 h and culture supernatant was collected. As a non-stimulated culture control, 24 h conditioned medium from saline (mock) treated cultures were also collected. The culture supernatants were clarified by centrifugation at 10,000 x g for 10 min and stored in single use aliquots at -80^o^ C.

To analyze the effect of IFNs on virus replication, pAECs were treated with serial dilutions of the poly I:C pAEC conditioned medium or control conditioned medium 8 h prior to infection with IAV at 0.01 MOI. After 1 h of virus adsorption, the inoculum was removed and the cells were washed twice with HBSS and allowed to grow in pAEC growth medium supplemented with same amount of conditioned medium. At 24 h p.i., culture medium was collected for analysis of virus titer by TCID_50_ assay.

### Statistical analysis

Results are presented as the mean ± SEM. A two tailed student t-test, assuming equal variance among samples, was used to determine statistical significant differences between the data sets using GraphPad Prism 6. An ANOVA with Dunnette’s post-test for comparisons to a common control was used for experiments comparing hTERT expression in primary, passage 15 and passage 35 pAECs. The distribution of sample values was tested using the method of Kolmogorov and Smirnov and found to follow a Gaussian distribution. A “p” value of less than 0.05 was considered significant.

## Results

### Characterization of primary and immortalized porcine airway epithelial cells (pAECs)

**Fig 1:**
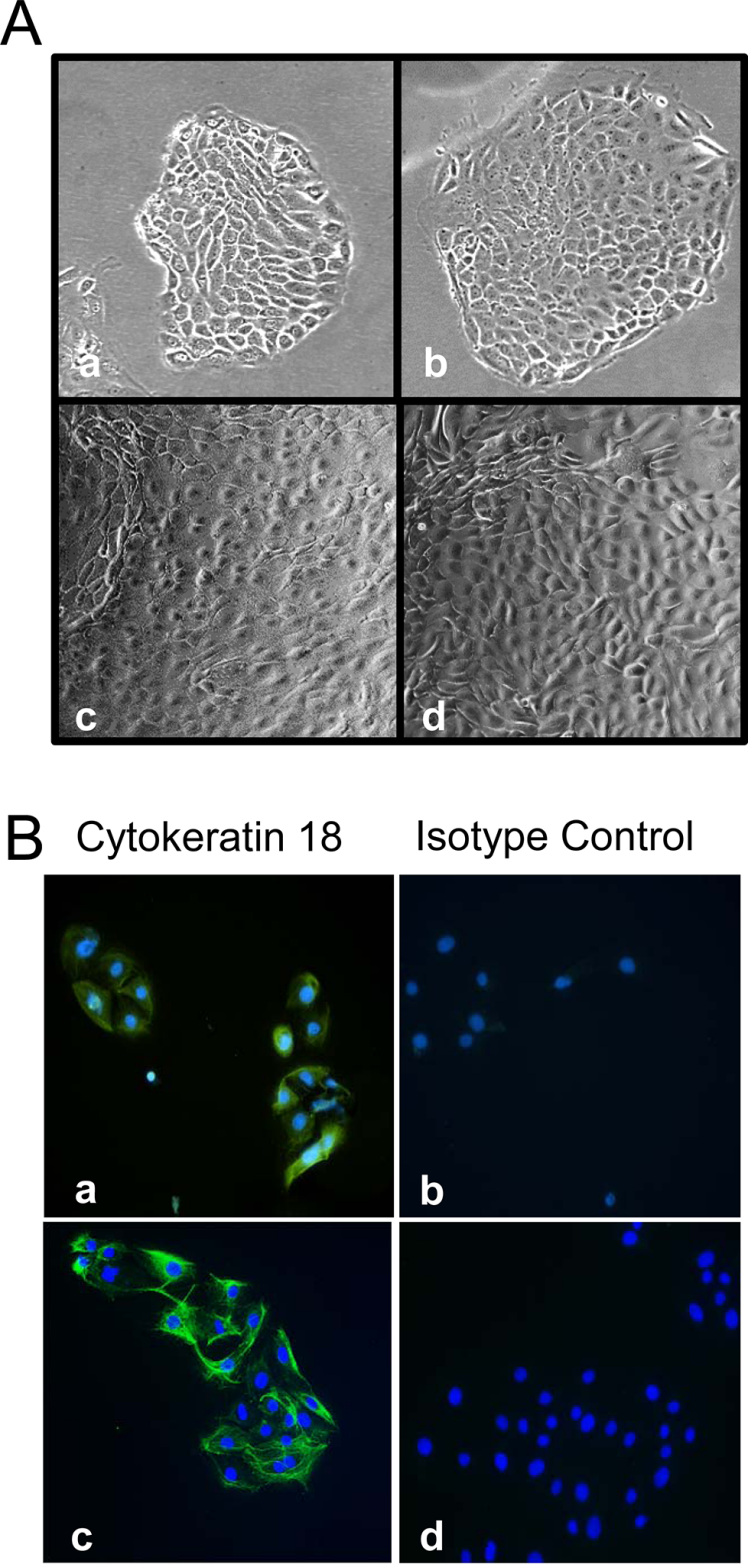
Morphological characterization of pAEC. (A) Primary pAECs (a and c) and immortalized pAECs (b and d) exhibit typical epithelial cell morphology and growth patterns. (B) Primary pAECs (a and b) and immortalized pAECs (c and d) were analyzed by immunofluorescence staining with anti-cytokeratin 18 (a and c) or isotype control (b and d). Nuclei were counter stained with DAPI. (Magnification 20X). The data are representative of three independent experiments.

**Fig 2:**
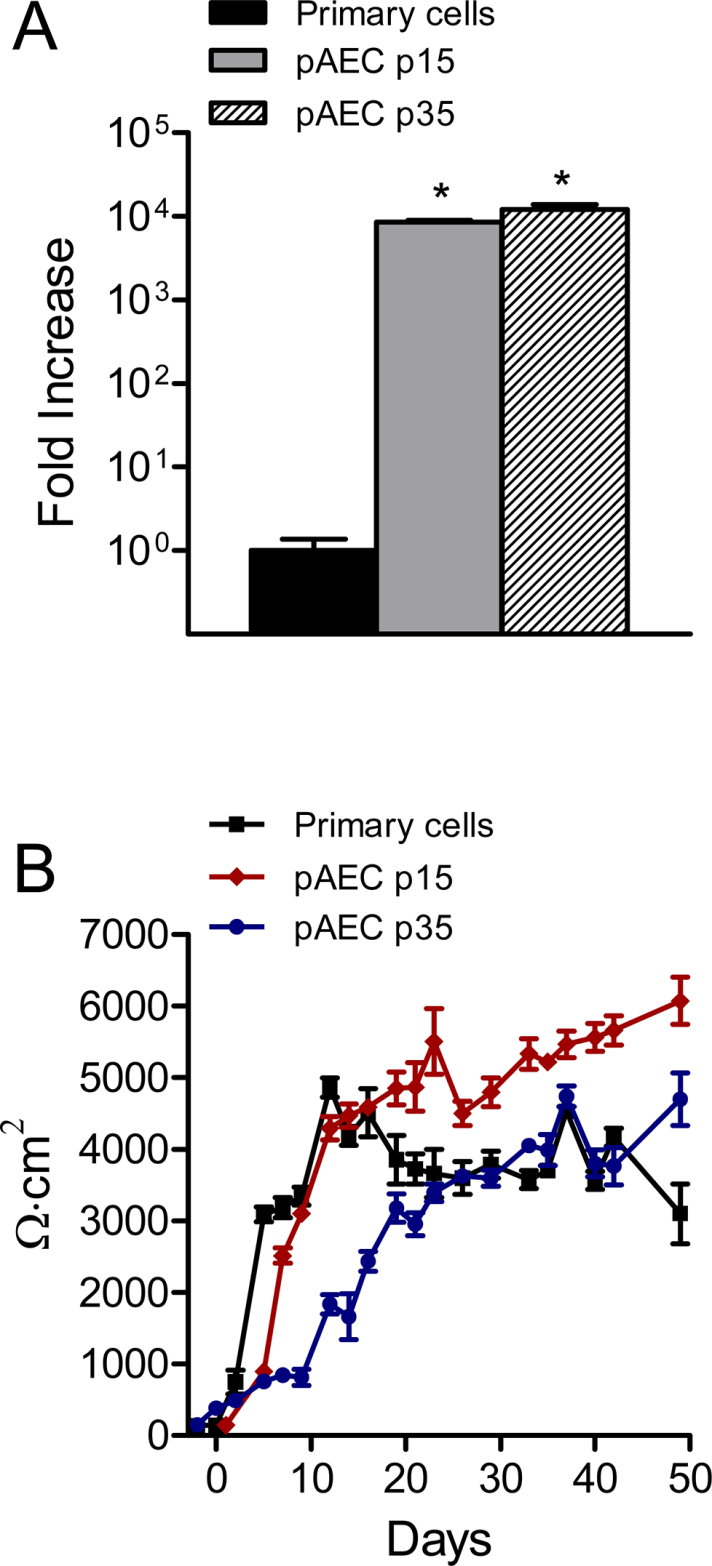
Telomerase expression and development of transepithelial resistance. (A) Normalized RT-qPCR data comparing levels of hTERT mRNA expression in primary, passage 15 and passage 35 pAECs (n = 6). Immortalized passage 15 and 35 cells had significantly higher levels of expression compared to primary cells (*p < 0.01). (B) Changes in transepithelial resistance (TER) over a time course of 50 days after seeding onto Snapwell^®^ membrane filters (n = 6 for each condition). Although passage 35 cells exhibited a slower increase in TER for the first 20 days, the steady-state TER was very similar to primary cells for the remaining 30 days.

pAECs displayed archetypal epithelial morphology as indicated in the phase-contrast images shown in Fig 1A. Additionally, pAECs immunofluorescence staining for cytokeratin, an epithelial marker, was performed using anti-cytokeratin 18 antibody. Both primary and immortalized pAECs (ipAECs) expressed cytokeratin 18 (Fig 1B) providing additional evidence for their identification as epithelial cells. Immortalization of pAECs was accomplished by constitutive expression of the human telomerase gene, hTERT in transfected cells. Expression of hTERT mRNA was confirmed by RT-qPCR and shown to be four orders of magnitude greater at passage 15 than in primary cells and no decrease in expression was observed at passage 35 (Fig 2A). To further characterize the epithelial character of pAECs and ipAECs, the time required for development of transepithelial electrical resistance was measured across cells grown on membrane filters under liquid-liquid interface conditions. Only epithelial cells are capable of polarization and the expression of tight junction complexes that give rise to high transepithelial resistances. In Fig 2B a comparison is presented of the development of TER over time in primary cells and ipAECs at passages 15 and 35. Note that all of the monolayers achieved comparably high steady-state resistances within 3 weeks after plating.

### Swine IAV grows more efficiently in pAECs than human pH1N1 CA/09

**Fig 3:**
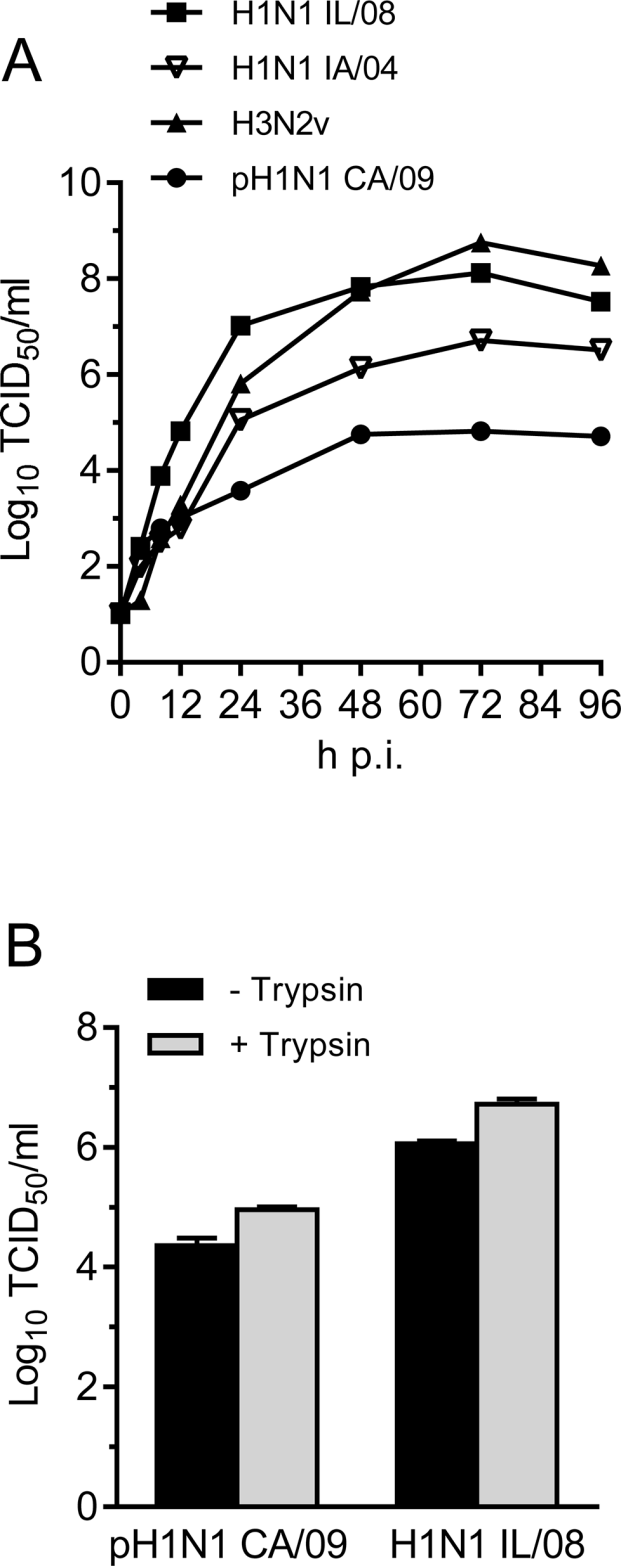
Replication of IAV in pAECs. (A) Primary pAECs were infected with the indicated virus at MOI of 0.01 without exogenous trypsin and the supernatants were collected at 0, 4, 8, 12, 24, 48, 72, and 96 h p.i.. Virus titers were determined by TCID_50_ assay in MDCK cells in the presence of 0.5 μg/ml TPCK-trypsin. (B) Titer of IAV pH1N1 CA/09 and H1N1 IL/08 in the culture supernatant of ipAECs 72 h p.i in the presence and absence of exogenous TPCK-trypsin. Data are average of 2 independent experiments (n = 2).

To determine the growth kinetics of IAV strains in primary pAEC, cells were infected at 0.01 MOI without exogenous trypsin. We observed that the swine IAV replicated effectively in primary pAECs, grew to higher titers compared to the human origin virus. Titers of swine H1N1 IL/08 were approximately 3 logs higher than human pH1N1 CA/09 at 24 h p.i. and remained higher until 96 h p.i. (Fig 3A). We further compared the growth of swine and human IAV in the ipAEC line and observed similar results with approximately 1.5 log higher titers for swine origin IAV compared to human origin IAV at 72 h p.i. (Fig 3B). Moreover, addition of TPCK-trypsin during infection of ipAECs did not significantly increase the virus titer, suggesting that the reduced growth of human influenza virus in pAECs was not due to inefficient activation of HA by host proteases.

### Swine H1N1 IL/08 and human pH1N1 CA/09 bind to pAECs with similar efficiency

**Fig 4:**
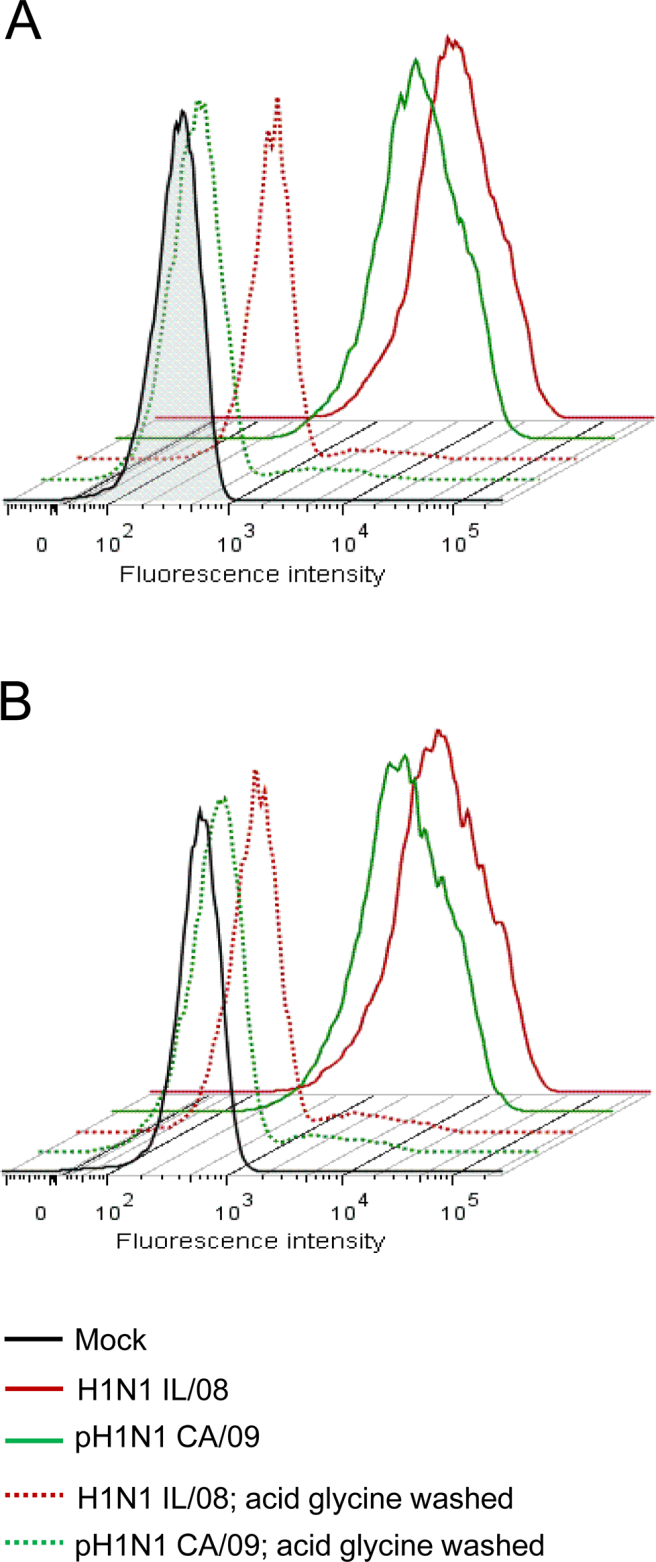
Binding of IAV to pAEC and ipAEC. Primary pAECs (A) or immortalized pAECs (B) were incubated with H1N1 IL/08 (red), pH1N1 CA/09 (green) or mock virus preparation (black) at an MOI of 50 for 90 min on ice. Cells were washed either with cold DMEM (solid lines) or acid glycine buffer (dotted lines). Surface bound virus was analyzed by flowcytometry using anti-NP monoclonal antibody followed by Alexa Fluor 647 conjugated goat anti-mouse IgG2a. Data are representative of three independent experiments.

To examine if the observed divergence in replication kinetics of swine and human origin IAV could be explained by differences in virus binding affinity to pAECs, we compared the ability of H1N1 IL/08 and human pH1N1 CA/09 IAV to bind to pAECs. H1N1 IL/08 was used as a representative swine origin virus for all experiments henceforth, given that this virus demonstrated the greatest difference in replication efficiency in pAECs compared to human pH1N1 CA/09. Primary pAECs and ipAECs were incubated with virus at 50 MOI and cell surface bound virus was identified using IAV NP specific monoclonal antibody, which recognizes all IAV strains. Flowcytometry analysis showed no significant differences between H1N1 IL/08 and human pH1N1 CA/09 IAV in their ability to bind to pAECs (Fig 4). In fact, the levels of cell surface virus was similar in both primary pAECs and ipAECs. In addition, washing virus bound cells with acid glycine buffer completely abrogated virus specific fluorescence back to levels in mock-infected cells, indicating that the virus detected by this method were bound to the cell surface and had not been internalized. These data suggest that differences in virus binding ability to cell surface receptors cannot explain the increased replication efficiency of swine H1N1 IL/08 in pAECs compared to pH1N1 CA/09.

### Induction of type I and type III IFNs was delayed by swine IAV in pAECs

**Fig 5:**
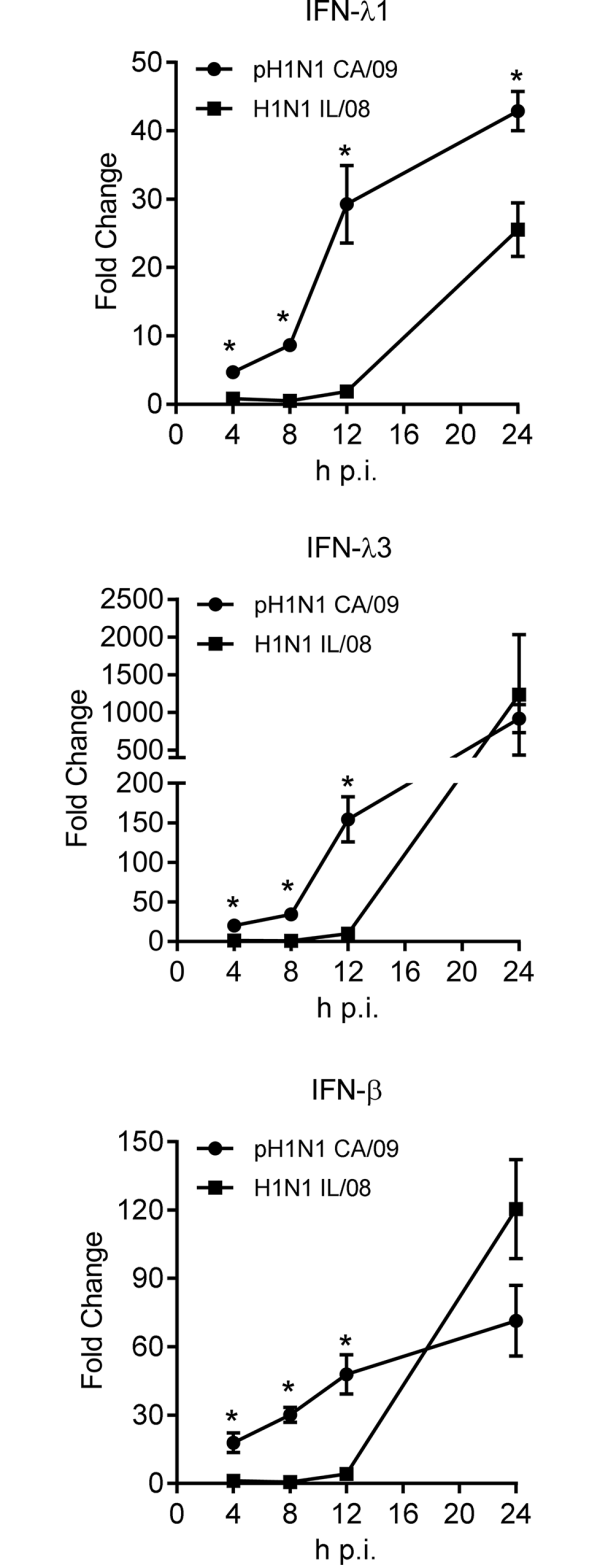
Kinetics of IFN gene expression in response to IAV infection of primary pAECs. Primary pAECs were infected with IAV pH1N1 CA/09 (circle) or H1N1 IL/08 (square) at 1 MOI and the total cellular RNA was extracted at indicated h p.i.. Interferon (IFN)- λ1, λ3, and β gene expression was analyzed by RT-qPCR. The ΔCT values were normalized to β-actin gene expression and represented as fold change (2^-ΔΔCT^) over mock infected cells. The experiment was performed in duplicate using cells derived from two donor animals. *p <0.05.

To determine if differences in replication kinetics of swine and human origin viruses in pAECs were due to differences in kinetics and magnitude of type I and type III IFN response to IAV, we assessed the ability of these viruses to induce expression of IFN transcripts in infected cells. Primary pAECs were infected with H1N1 at 1 MOI and kinetics of IFN gene expression in response to IAV was determined by measuring levels of IFN-α, IFN-β, IFN-λ1, and IFN- λ3 genes at 4, 8, 12, and 24 h p.i. Induction of IFN-β and IFN-λ genes were observed in human pH1N1 CA/09 infected pAECs as early as 4 h p.i. (Fig 5). In contrast, induction of IFN expression was delayed up to 12 h p.i. in swine H1N1 IL/08 infected cells. At 12 h p.i. the level of IFN-β, IFN-λ1 and IFN-λ3 gene transcripts were 10 to 15 fold higher in pAECs infected with pH1N1 CA/09 compared to H1N1 IL/08. However, the magnitude of induction of IFN-β and IFN-λ3 at 24 h p.i. was not significantly different between the swine and human origin influenza viruses tested. Consistent with the reported findings [27, 28] no significant induction of IFN-α was observed by both these viruses examined (data not shown). Taken together, these results suggest that the delay in induction of IFN-β and IFN-λ by swine H1N1 IL/08 allow the virus to spread rapidly leading to higher titers in pAEC cultures.

### Swine and human isolates of IAV differ in their sensitivity to the poly I:C induced IFN response in pAECs

**Fig 6:**
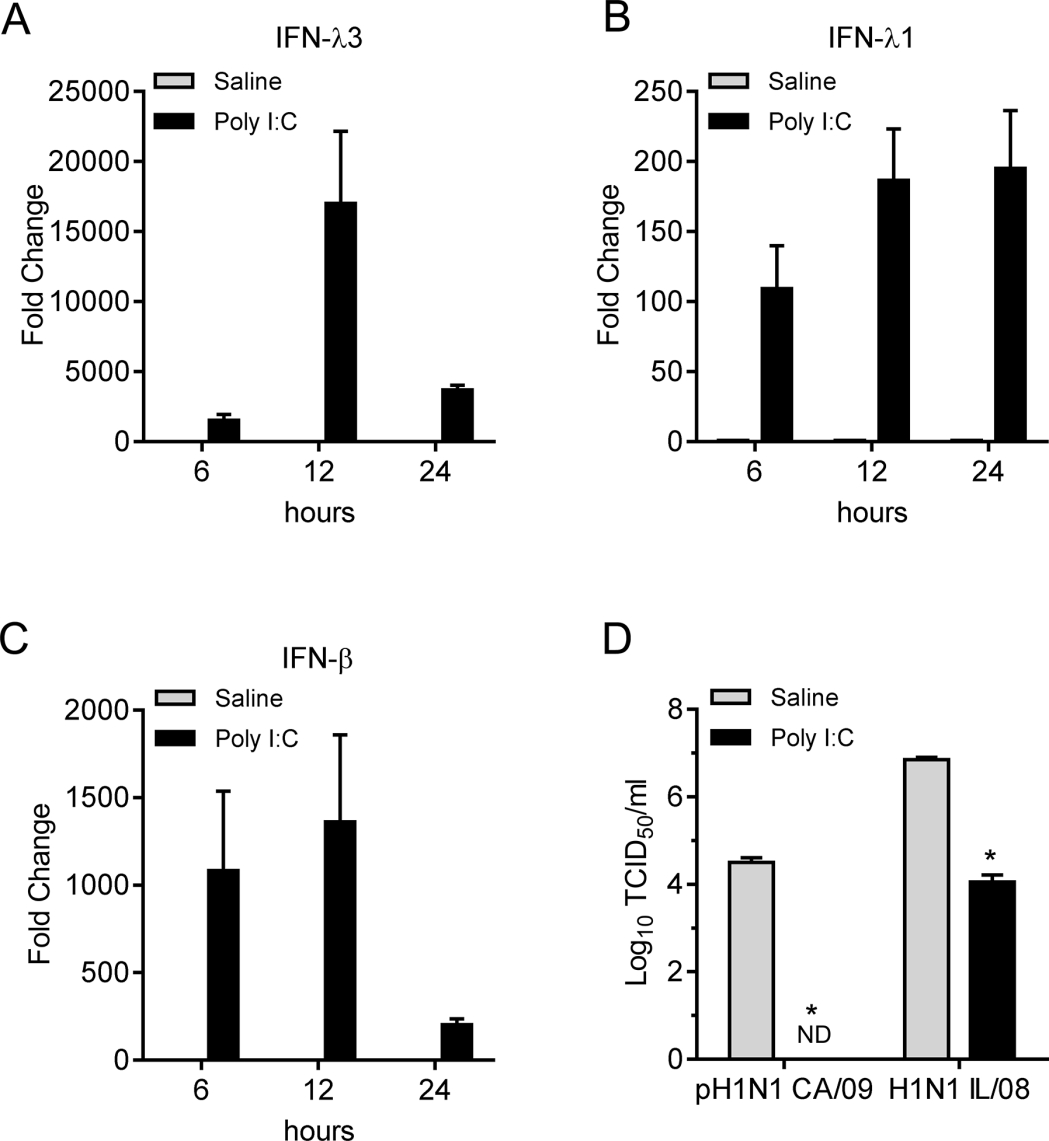
Resistance of swine and human origin IAV strains conferred to poly I:C induced innate response. (A-C) Analysis of IFN-λ3 (A), IFN-λ1 (B), and IFN-β (C) gene expressions in response to poly I:C treatment by primary pAECs is presented. Cells were treated with 50 μg/ml poly I:C and the total cellular RNA was extracted at indicated time post treatment. Interferon gene expression was analyzed by RT-qPCR. Data were normalized to β-actin gene expression and represented as fold change over saline (vehicle) treated cells. The experiment was performed in duplicate using cells derived from two donor animals. (D) Titers of IAV pH1N1 CA/09 and H1N1 IL/08 in pAECs pretreated with poly I:C or saline (vehicle) for 24 h prior to infection (0.01 MOI). Virus titers in culture supernatants were assessed 24 h p.i. by TCID_50_ assay. Data are average of two independent experiments performed in duplicate. ND; not detected. *p <0.05.

We next investigated if swine and human IAV differ in their ability to counteract the effect of interferons produced by pAECs. To induce type I and type III IFNs, pAECs were treated with 50μg/ ml poly I:C, a synthetic analogue of dsRNA, 24 h prior to infection. Treatment of pAECs with poly I:C induced IFN-β, IFN- λ1, and IFN- λ3 gene transcripts, which peaked at 12 h post treatment (Fig 6A–6C). There was approximately 3 log reduction in the titer of both H1N1 IL/08 and pH1N1 CA/09 at 24 h p.i. when pAECs were pretreated with poly I:C 24 h prior to infection (Fig 6D). Titers of the human origin strain, pH1N1 CA/09, were below detectable limits of the TCID_50_ assay.

**Fig 7:**
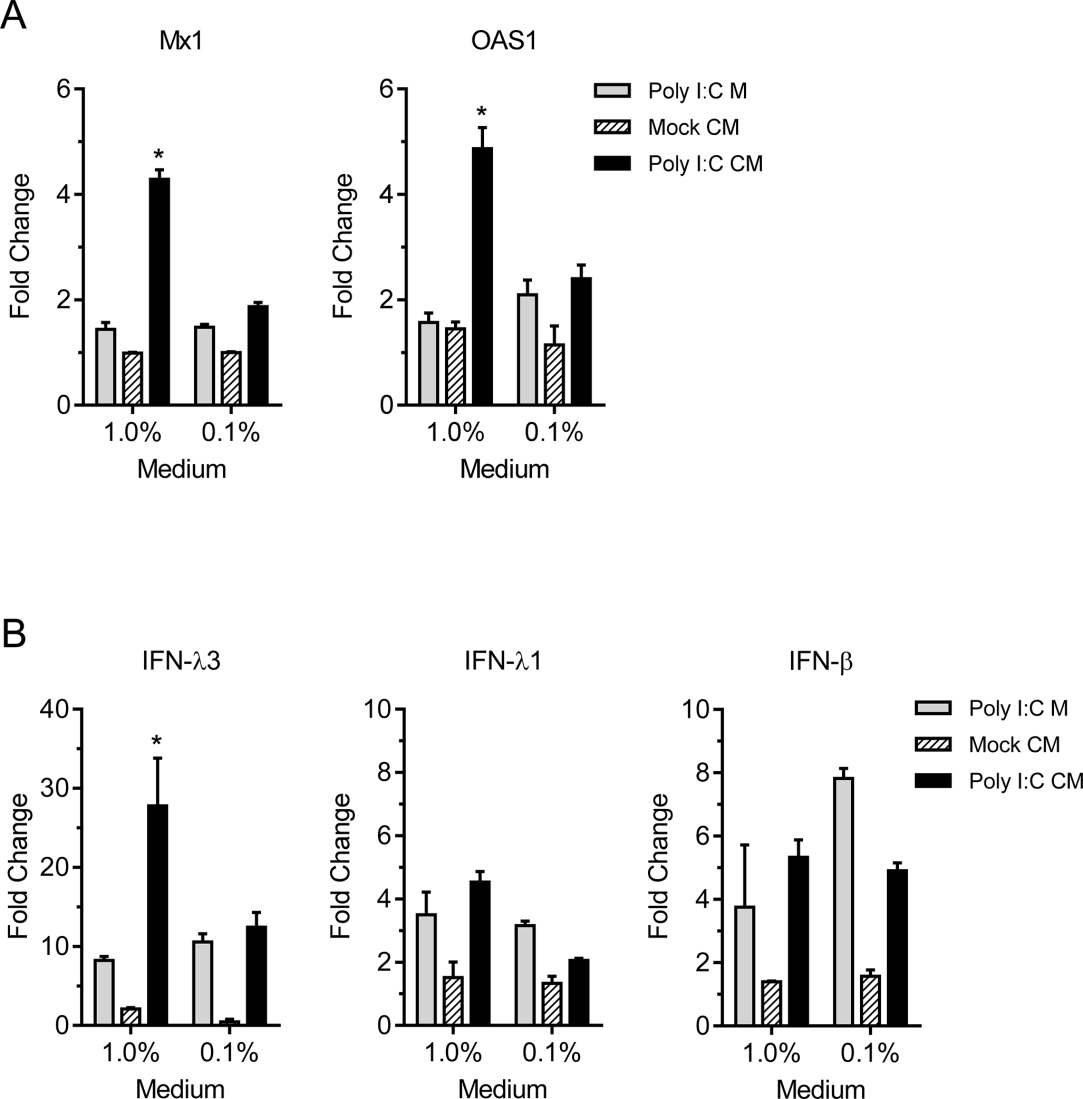
Conditioned medium from Poly I:C stimulated cultures increases ISG and IFN-λ3 expression in pAECs. Conditioned medium (CM) was prepared by treating pAECs for 24 h with 50 μg/ml poly I:C (poly I:C CM) or with saline (mock CM) and diluted in pAEC growth medium at indicated concentrations. Control medium was prepared by adding poly I:C (50 μg/ml) in pAEC growth medium (poly I:C M) and similarly diluted to compare the direct effect of residual poly I:C in the conditioned medium. pAECs were treated at indicated concentration of conditioned medium for 12 h. (A) Analysis of ISG expression in pAECs treated with conditioned medium from poly I:C treated cells. Total RNA was analyzed for the expression of Mx1 and OAS1 by RT-qPCR. Fold change in gene expression over growth medium and normalized to β-actin is presented. (B) Analysis of IFN expression in pAEC treated with conditioned medium (Poly I:C CM), Mock CM, or control medium (Poly I:C M), as described above, demonstrating fold change in expression of IFN-λ3, IFN-λ1 and IFN-β genes over growth medium and normalized to β-actin. *p <0.05.

**Fig 8:**
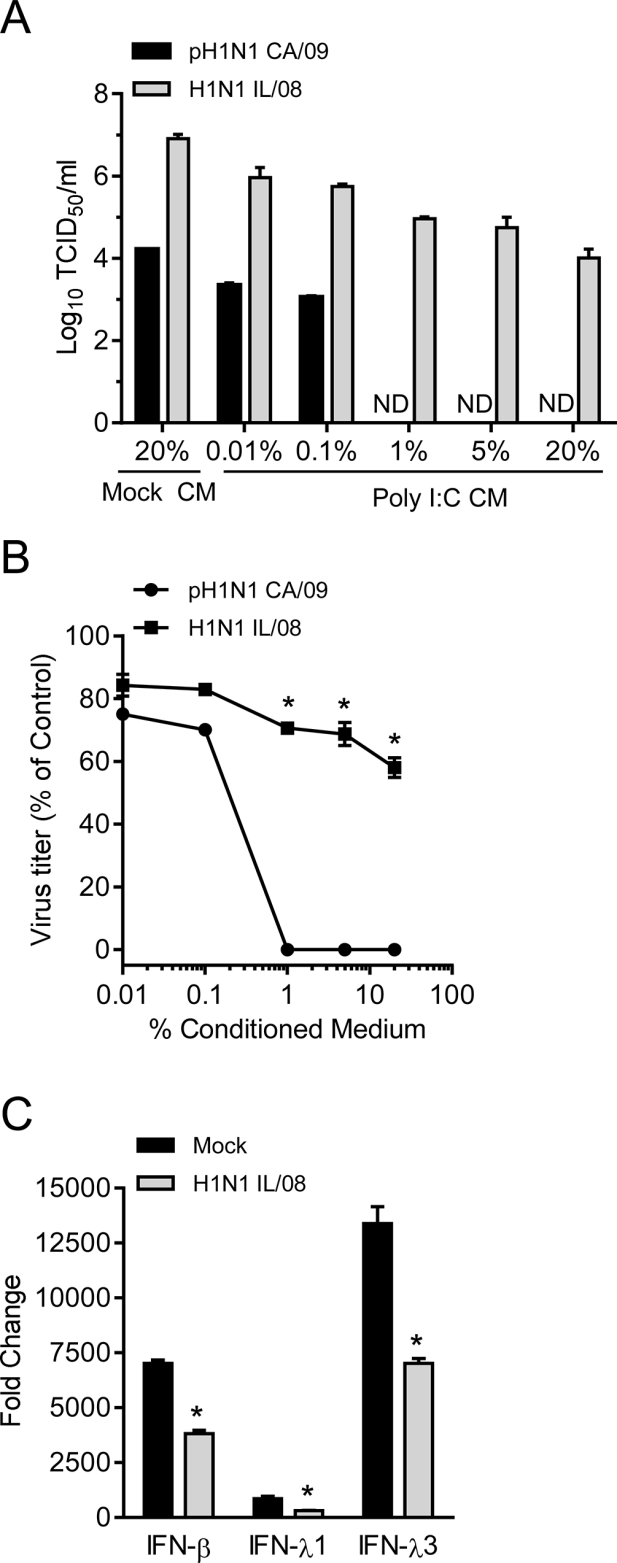
Conditioned medium from Poly I:C stimulated cultures differentially inhibits IAV replication in pAECs. (A) Titers of IAV pH1N1 CA/09 and H1N1 IL/08 in pAECs pretreated with conditioned medium. Cells were treated with indicated concentrations of poly I:C conditioned medium (poly I:C CM) or mock CM for 8 h prior to infection with IAV (0.01 MOI) as described in Materials and Methods. Culture supernatant collected 24 h p.i. were assessed by TCID_50_ assay on MDCK cells. Data is expressed as the mean ± SEM of two independent experiments performed in duplicate. ND; not detected. (B) The percent reduction in virus titer was calculated from data in (A) and expressed as percent of mock CM treated control. (C) Analysis of IFNs in pAECs treated with poly I:C 1 h after infection with H1N1 IL/08. pAECs were infected with H1N1 IL/08 at 3 MOI and 50 μg/ml poly I:C was added 1 h p.i. Total RNA was extracted at 12 h p.i. and the expression of IFNs was analyzed by RT-qPCR. Data are presented as the mean ± SEM of two independent experiments performed in triplicate. *p <0.05.

To further assess the effect of poly I:C induced innate response on virus replication, pAECs were treated with conditioned medium from poly I:C treated pAECs for 8 h before infection. 1% conditioned medium was sufficient to induce significantly higher expression levels of IFN stimulated genes, Mx1 and OAS1, 12 h post treatment compared to stimulation with similarly diluted poly I:C containing medium or conditioned medium from saline treated cells (Fig 7A). These data suggest the presence of secreted IFNs in conditioned medium of poly I:C treated pAECs is sufficient to induce ISGs in uninfected cells. In addition, conditioned medium of poly I:C treated pAECs also induced significant higher levels of IFN-λ3 gene expression (Fig 7B), consistent with the previous report that IFN-λ was induced by type I and type III IFNs [29]. Pretreatment of pAECs with conditioned medium from poly I:C treated cells inhibited growth of both pH1N1 CA/09 and H1N1 IL/08 in a concentration dependent manner (Fig 8A). However, the magnitude of titer reduction was significantly higher with pH1N1 CA/09 infection than with swine origin H1N1 IL/08 (Fig 8B). These data indicate that the swine H1N1 IL/08 is less sensitive to the innate antiviral effect of poly I:C compared to pH1N1 CA/09 in swine airway epithelial cells.

To determine if the infection of pAECs with H1N1 IL/08 inhibits poly I:C induced IFN-β and IFN-λ gene expression, we stimulated primary pAECs with 50 μg/ml poly I:C 1 h after infection at 3 MOI. Analysis of IFN gene expression 12 h p.i. showed that the H1N1 IL/08 infection significantly reduced poly I:C induced IFN-β and IFN- λ expression in pAECs compared to mock infection (Fig 8C). These data indicate that swine H1N1 IL/08 is not only less sensitive to the effect of IFNs than human pH1N1 CA/09 but was also suppressed the induction of IFNs in swine epithelial cells.

## Discussion

Establishment of influenza virus infection in the respiratory tract relies on several factors, including availability of receptors on the host cell surface as well as the innate antiviral responses elicited by host cells. Primary swine airway epithelial cells have been used previously as an *in vitro* model to study IAV infection [5, 6, 27]. These cells serve as a useful model to study IAV replication and pathogenesis including host innate responses, since IAV targets epithelial cells of the respiratory tract and these cells are the key contributor to innate antiviral responses via production of IFNs. In addition, immortalized pAECs described here exhibited properties similar to primary pAECs and are susceptible to IAV infection without exogenous trypsin.

In the present study, we observed differences in infectivity of human and swine IAV in swine airway epithelial cells. The human strain, pH1N1 CA/09 replicated less efficiently compared to swine IAV H1N1 IL/08, H1N1 IA/04, and H3N2v in primary pAECs. Attachment of the virus to the host cell surface is critical for tissue tropism of virus infections. Since IAV attachment to sialic acid containing receptor on the host cell surface is mediated by haemagglutinin (HA), this viral protein is considered an important determinant for virus tropism [5, 6, 30]. Previous studies showed that the human and swine IAV preferentially bind to α2,6,-linked sialic acid containing receptors [6, 31, 32] and the binding specificity can be changed by a single amino acid substitution in viral HA protein [30, 33]. Most avian influenza viruses bind to α2,3,- linked sialic acid receptor [34]. Swine respiratory tract consists of both α2,6,- and α2,3,- linked sialic acid containing receptors [2, 5], although α2,6,-linked receptors are more abundant in upper respiratory tract [35]. Both human and swine IAV bind to α2,6-linked sialic acid receptors with preference for N-acetylneuraminic acid (NeuAc) over N-glycolylneuraminic acid (NeuGc) [32]. However, virus binding studies using glycan microarray indicated that viruses that bind to both biantennary and polylactosamine glycans have high infectivity compared to viruses that bind only to polylactosamine glycans [6]. Recent studies using shotgun glycomics of pig lung with a panel of influenza viruses isolated from humans, birds, and swine showed differences in endogenous N-glycan recognition for each virus [36]. While it is likely that the amino acid differences in the HA protein and consequent differences in virus binding contribute to the differences in infectivity of human and swine IAV in pAECs, our results showed no significant difference in levels of binding of both H1N1 IL/08 and pH1N1 CA/09 to pAECs.

IFNs have been shown to play a critical role in limiting IAV infection and transmission [37, 38]. IFNs function in an autocrine and paracrine manner inducing anti-viral state in both infected and surrounding uninfected cells. Interestingly, we found the swine H1N1 IL/08, which replicated faster and to higher titers in primary pAECs induced significantly lower levels of IFN-β, IFN-λ1 and IFN-λ3 during the first 12 h p.i. compared to pH1N1 CA/09, which rapidly induced IFN genes as early as 4 h p.i. However, the magnitude of expression of IFN genes at 24 h p.i. was not significantly different between the two viral strains examined. This indicates a delay in induction of IFNs by swine H1N1 IL/08 may have resulted in higher titer in pAECs. This observation is in agreement with the recent report showing that the ability of H5N1 viruses to prevent human IFN-β induction enhances replication in human epithelial cells [39] Similarly, delayed antiviral signaling and impaired type I and type III INF induction by pathogenic human influenza H3N2 compared to low pathogenic avian influenza H11N9 resulted in more efficient replication of H3N2 in Calu-3 cells [40].

IFN responses differ among different cell types and in different hosts. Expression of type I and type III IFNs in IAV infected epithelial cells or in precision cut lung slices have been studied previously [27, 28]. Recently it has been demonstrated that the IFN-β and IFN-λ gene expression following IAV infection or 24h treatment with poly I:C was lower in bronchial epithelial cells of adult swine compared to that of human, although both cell types induced IFN response [27]. We observed substantial levels of IFN-β, IFN-λ1 and IFN-λ3 gene expression in poly I:C treated primary pAECs derived from neonatal pigs with the peak induction at ~12 h post treatment. However at 24 h post treatment there was significant drop in the expression level of IFN-β and IFN-λ3. Pretreatment of pAECs with poly I:C resulted in approximately 3 log reduction in growth of both pH1N1 CA/09 and H1N1 IL/08. Since the growth of pH1N1 CA/09 was less efficient even in saline treated control cells, the titer of this virus in poly I:C pretreated cells was reduced below the limit of detection of our TCID_50_ assay. In addition, treatment of pAECs with conditioned medium from poly I:C treated cells, which stimulated Mx1 and OAS1 genes, reduced the replication of both pH1N1 CA/09 and H1N1 IL/08 in a concentration dependent manner. However, H1N1 IL/08 was less sensitive to the dsRNA induced antiviral effect of swine cells compared to pH1N1 CA/09 as indicated by the relatively lower percentage of reduction in virus titer. Our results also demonstrated that IFN response induced by poly I:C in pAECs was reduced by the swine H1N1 IL/08 infection at 12 h p.i. IAV are known to possess mechanisms to counteract IFN mediated innate immunity. IAV NS1 protein is an effective type I IFN antagonist, which acts by multiple mechanisms [17–23]. Furthermore, NS1 from pathogenic human influenza H3N2 has been shown to inhibit induction of type I and type III IFNs more effectively than low pathogenic avian influenza H11N9 strain [40]. It is possible that NS1 of H1N1 IL/08 is more effective in inhibiting IFN induction than pH1N1 CA/09, but further work is required to fully address this hypothesis.

Collectively, our data showed that the human pH1N1 CA/09 and swine H1N1 IL/08 differ in their ability to induce and respond to type I and type III IFNs in porcine cells. Swine origin IAVs may have evolved for better adaptation in swine populations presumably by subverting host innate responses to infection. The rapid induction of IFN-β and IFN-λ genes by human pH1N1 CA/09 in infected pAECs suggest that this virus is less adapted to swine cells. Thus, subverting early innate antiviral responses may be an important strategy for influenza viruses to cross the species barrier and adapt to the new host. Further studies evaluating the ability of other human IAV strains to induce type I and type III interferons in pAECs would help to understand the innate immune response of pigs to human IAV and their mechanisms of regulation.
